# A novel orally active HDAC6 inhibitor T-518 shows a therapeutic potential for Alzheimer’s disease and tauopathy in mice

**DOI:** 10.1038/s41598-021-94923-w

**Published:** 2021-07-29

**Authors:** Tomohiro Onishi, Ryouta Maeda, Michiko Terada, Sho Sato, Takahiro Fujii, Masahiro Ito, Kentaro Hashikami, Tomohiro Kawamoto, Maiko Tanaka

**Affiliations:** 1 Neuroscience Drug Discovery Unit, Research, Takeda Pharmaceutical Company Limited, 26-1, Muraoka-Higashi 2-chome, Fujisawa, Kanagawa 251-8555 Japan; 2 Drug Metabolism and Pharmacokinetics Research Laboratories, Research, Takeda Pharmaceutical Company Limited, 26-1, Muraoka-Higashi 2-chome, Fujisawa, Kanagawa 251-8555 Japan; 3 Discovery Biology, Discovery Science, Axcelead Drug Discovery Partners, Inc., 26-1, Muraoka-Higashi 2-chome, Fujisawa, Kanagawa 251-0012 Japan

**Keywords:** Biochemistry, Enzymes, Proteins

## Abstract

Accumulation of tau protein is a key pathology of age-related neurodegenerative diseases such as Alzheimer's disease and progressive supranuclear palsy. Those diseases are collectively termed tauopathies. Tau pathology is associated with axonal degeneration because tau binds to microtubules (MTs), a component of axon and regulates their stability. The acetylation state of MTs contributes to stability and histone deacetylase 6 (HDAC6) is a major regulator of MT acetylation status, suggesting that pharmacological HDAC6 inhibition could improve axonal function and may slow the progression of tauopathy. Here we characterize *N*-[(1R,2R)-2-{3-[5-(difluoromethyl)-1,3,4-oxadiazol-2-yl]-5-oxo-5H,6H,7H-pyrrolo[3,4-b]pyridin-6-yl}cyclohexyl]-2,2,3,3,3-pentafluoropropanamide (T-518), a novel, potent, highly selective HDAC6 inhibitor with clinically favorable pharmacodynamics. T-518 shows potent inhibitory activity against HDAC6 and superior selectivity over other HDACs compared with the known HDAC6 inhibitors in the enzyme and cellular assays. T-518 showed brain penetration in an oral dose and blocked HDAC6-dependent tubulin deacetylation at Lys40 in mouse hippocampus. A 2-week treatment restored impaired axonal transport and novel object recognition in the P301S tau Tg mouse, tauopathy model, while a 3-month treatment also decreased RIPA-insoluble tau accumulation. Pharmaceutical inhibition of HDAC6 is a potential therapeutic strategy for tauopathy, and T-518 is a particularly promising drug candidate.

## Introduction

Axon plays an important role in the bidirectional transport of various nutrient factors, growth factors, neurotransmitters, and even whole organelles between the soma and synapse in neurons. Axon consists of microtubules (MTs), and axonal stability^1,2^ and transport^3,4^ is enhanced by Lys40 acetylation of MT. As Histone deacetylase 6 (HDAC6) is a major regulator of MT Lys40 acetylation status^5,6^ and is not a deacetylase for Histone unlike other HDACs (e.g. HDAC1–3), HDAC6 selective inhibition is considered a potential therapeutic target that enhances axonal function. Axonal dysfunction and degeneration are observed in many neurodegenerative diseases including Alzheimer's disease (AD), Parkinson's disease (PD), and amyotrophic lateral sclerosis (ALS)^7,8^.

In AD, axonopathy and white matter alterations are observed from the early stage prior to the onset of severe cognitive symptoms^9–11^, while in the later disease stage, increase of cerebrospinal fluid (CSF) neurofilament light, a biomarker for axonal damage, is correlated with cognitive dysfunction and tau pathology^12–14^. In addition to AD, axonal damage is associated with the severity and progression of other neurodegenerative diseases such as frontotemporal degeneration^15,16^. In these diseases, tau accumulation is commonly observed in the brain and they are collectively termed tauopathy. Tau accumulation is linked to disruption of MTs and following axonal degeneration because tau is a MT-binding protein and regulates axonal function^17^. Conversely, the changed structure of MT could accelerate intracellular tau accumulation by altering subcellular localization of tau from MT to cytosol. Today, there are no disease-modifying therapies that slow or halt disease progression in tauopathies. Drugs able to prevent tau accumulation and ensuing axonal degeneration are promising therapeutic candidates for the treatment of tauopathies.

Several studies have found elevated HDACA6 expression^18–20^ or reduced acetylated tubulin^21,22^ in the brains of AD. Therefore, HDAC6 inhibitor could prevent axonal dysfunction, thereby slowing tauopathy progression. In fact, several HDAC6 inhibitors have been reported to increase axonal transport of mitochondria and neurotrophic factors in various disease cell models^4,23–25^ and Tubastatin A, ACY-1215, MPT0G211, and 5-Aroylindoles have been demonstrated to have therapeutic potential including reduction in tau pathology in animal models of AD and other tauopathies^26–29^.

Inhibition of HDAC is dependent on a zinc-binding group (ZBG) that interacts with the HDAC catalytic domain. In most reported HDAC6 inhibitors including the molecules described above, the ZBG is hydroxamate, which may cause suboptimal pharmacokinetics, non-selective interaction such as hERG cardiac ion channel inhibition, and mutagenicity^30^. Moreover, hydroxamate-based HDAC inhibitors show off-target neuroprotective effects by forming antioxidant metal complexes rather than via HDAC inhibition per se^31,32^. Therefore, a non-hydroxamate HDAC6 inhibitor would be preferable to precisely evaluate the therapeutic potential of pharmacological HDAC6 inhibition. Recently, oxadiazole-type ZBG have been invented as a novel class of HDAC6 inhibitors^33^. Here, we report that the novel HDAC6 inhibitor T-518 harboring oxadiazole instead of hydroxamate as the ZBG acts as a potent, selective, orally bioavailable, and brain-penetrant HDAC6 inhibitor, and that T-518 has therapeutic effects in a mouse model of tauopathy.

## Results

### Discovery and in vitro profiling of T-518

**Figure 1:**
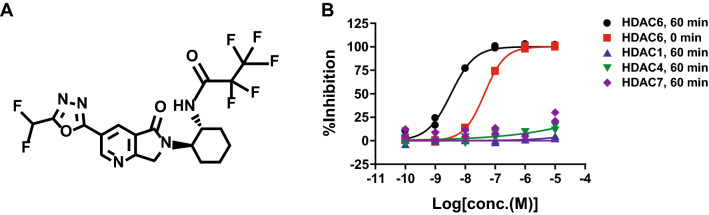
Chemical structure of T-518 and enzyme inhibition curve for HDAC6, 1, 4, and 7.

We discovered a novel and potent HDAC6 inhibitor T-518 through an intensive medicinal chemistry optimization of high-throughput screening hit (Fig. 1A)^34^. T-518 has a unique chemical structure while most known HDAC6 inhibitors harbour the hydroxamate. T-518 inhibited human HDAC6 enzyme activity with IC50 values of 36 nM (without pre-incubation) and 4.6 nM (after 60 min of pre-incubation) (Fig. 1B). In contrast, concentrations up to 10 μM did not obviously inhibit human HDAC1, 4, or 7, which belongs to Class I or Class IIa HDAC.

**Figure 2:**
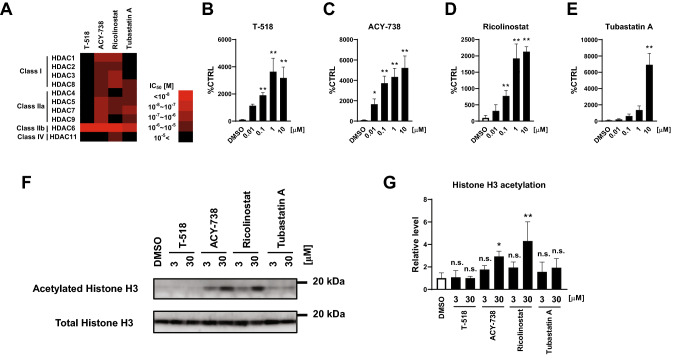
Selectivity profiling of HDAC6 inhibitors. Heat map summary of HDAC inhibition is shown (**A**). T-518 and hydroxamate HDAC inhibitors ACY-738, Ricolinostat, and Tubastatin A were treated to DIV6 mouse primary neural culture at 0.1–10 μM for assessment of tubulin acetylation (**B**–**E**) and at 3 and 30 μM for and H3 acetylation (**F**,**G**), respectively. Twenty-four hours after treatment, cell lysates were extracted with RIPA and tubulin acetylation was examined by ELISA and H3 acetylation was by Western blotting. Full-length blot of F is presented in Figure S5. Data are expressed as mean ± SD and analyzed statistically as follows: Williams test (*p < 0.05, **p < 0.01 in **B**–**E**), Dunnet test (**p < 0.01 in **G**). n = 5. *n.s.* not significant.

In addition to the screening assay, HDAC selectivity profile of T-518 was evaluated by HDAC panel and compared with other well-known hydroxamate HDAC6 inhibitors such as ACY-738, Ricolinostat, and Tubastatin A (Figure S1). Result represented by the heat map shows T-518 has high selectivity over other HDACs (Fig. 2A). To examine the effect of T-518 and other known HDAC6 inhibitors in cells, mouse primary neural cultures were treated with T-518, ACY-738, Ricolinostat, and Tubastatin A, or DMSO for 24 h and effects on tubulin acetylation and Histone H3 acetylation were evaluated. Every inhibitor significantly and concentration-dependently elevates tubulin acetylation (Fig. 2B–E), consistent with enzyme activity assay. ACY-738 and Ricolinostat also increase the acetylation of histone H3, while neither T-518 nor Tubastatin A at 3 and 30 μM demonstrates this activity (Fig. 2F,G and Figure S5). These findings suggest that selectivity for other HDACs such as class I can alter H3 acetylation and T-518 is highly selective for HDAC6 both in both cell free system and mammalian neurons.

### In vivo pharmacokinetics (PK) and pharmacodynamics study of T-518

**Figure 3:**
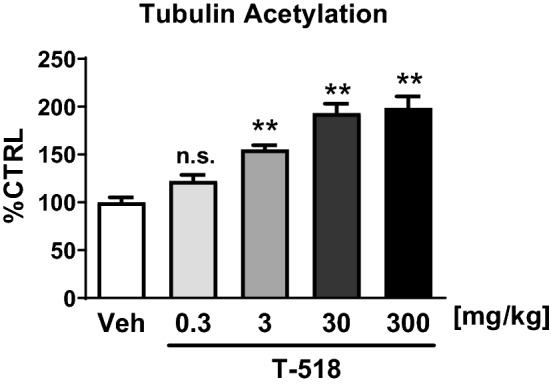
In vivo pharmacodynamics change of T-518. T-518 was administered orally at 0.3, 3, 30, and 300 mg/kg to 8-week-old male C57BL/6J mice. At 4 h after administration, the hippocampus was dissected and acetylated tubulin was evaluated by ELISA. Data are expressed as mean ± SEM and analyzed statistically as a follow: two-tailed Williams test (**p < 0.01), n = 6. *n.s.* not significant.

**Figure 4:**
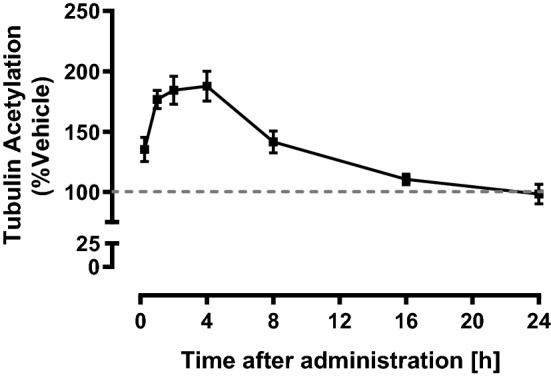
Time course study of pharmacodynamics of T-518. The compound was orally administered to 10-week-old male C57BL/6J mice at 3 mg/kg, and effect on tubulin acetylation in hippocampus was measured at 0.25, 1, 2, 4, 8, 16, and 24 h post-treatment. Data are expressed as mean ± SEM, n = 5. Statistical analysis was not performed.

To evaluate tubulin acetylation by T-518 in vivo, T-518 was orally administered at 0.3–300 mg/kg in mice and acetylated tubulin in the brain was assessed. T-518 dose-dependently increased tubulin acetylation in hippocampi 4 h after administration (Fig. 3). The time course of T-518 biodistribution in plasma and brain tissue (Table 1) as well as the effects on brain tubulin acetylation (Fig. 4) were then examined. Briefly, 3 mg/kg was administered orally to mice, and samples of hippocampus and plasma were collected 0.25, 1, 2, 4, 8, 16, and 24 h later. Tubulin acetylation increased progressively post-administration, reaching a plateau within 1–4 h and then gradually returning to the control level (Fig. 4). The concentration of T-518 in the hippocampus and plasma were measured and C_max_, T_max_, AUC, and MRT were calculated (Table 1). The concentration in the hippocampus was 12‒14% of that in plasma in C_max_ and AUC and exceeded the IC_50_. We cannot rule out that the hippocampal concentrations contain blood-derived fraction since no prior perfusion was performed when the hippocampus was sampled in this study. However, the concentration ratio of hippocampus to plasma was at least four times higher than volume fraction of blood in mouse brain, which is 3% based on the literature^35^. Thus, the dose administered was sufficient for substantial HDAC6 inhibition.

**Table 1:** Pharmacokinetic (PK) parameter of T-518 in C57BL mice at 3 mg/kg, po.

Matrices	PK parameters	Value	
Plasma	C_max_	1037	[ng/mL]
	T_max_	0.25	[h]
	AUC_0–24 h_	1722.5	[ng·h/mL]
	MRT	1.76	[h]
Hippocampus	C_max_	144	[ng/g]
	T_max_	0.25	[h]
	AUC_0–24 h_	202	[ng·h/g]
	MRT	1.33	[h]

Data are expressed as mean. n = 5.

**Figure 5:**
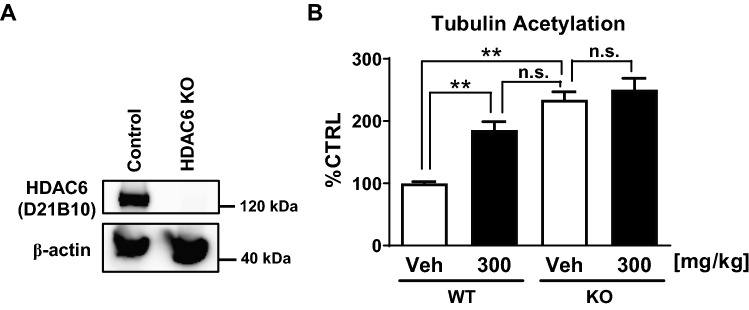
HDAC6 dependency of in vivo pharmacodynamics effect of T-518. Absence of HDAC6 expression in the cerebral cortex of HDAC6 KO mice was confirmed by Western blotting (**A**). T-518 was administered to 27-week-old male WT mice or HDAC6 KO mice at 300 mg/kg, and tubulin acetylation was assessed (**B**). Full-length gel of (**A**) is presented in Figure S6. Data was expressed as mean ± SEM and analyzed statistically as a follow: Tukey test (**p < 0.01), n = 5. *n.s.* not significant.

Next, to confirm that effect on tubulin acetylation is dependent on HDAC6 inhibition, T-518 was examined in wild type and HDAC6 KO mice. We developed HDAC6 KO mice and confirmed that expression of HDAC6 was completely absent in KO mouse brain (Fig. 5A). There is no significant change in body weights between KO and littermate WT (Figure S2B), and no abnormality in normal behavior and development is observed in the KO mice. As expected, administration of 300 mg/kg T-518 significantly elevated tubulin acetylation in WT mice but had no effect on HDAC6 KO mice (Fig. 5B and Figure S6), suggesting that the effect is dependent on HDAC6 inhibition.

### Improvement of axonal transport by T-518 in a tauopathy mouse model

**Figure 6:**
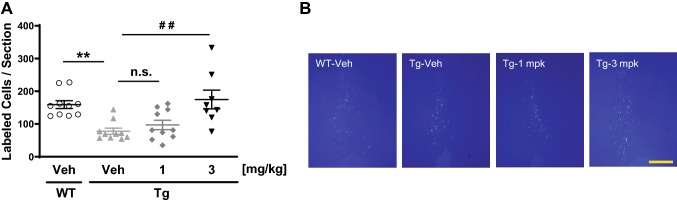
Rescue of impaired septohippocampal retrograde axonal transport in P301S tau Tg mice by T-518. Five-month-old WT and P301S tau Tg mice received 1 and 3 mg/kg T-518 or vehicle daily for 2 weeks, followed by injection of retrograde axonal tracer Fluoro-Gold into right hippocampus. Three days later, labeled neurons in the medial septum were counted in vehicle-treated, vehicle-treated Tg, and T-518-treated Tg (**A**). Representative image of the septal nucleus in each group was shown in (**B**). Data are expressed as mean ± SEM and as scatter plots, and analyzed statistically as follows: Student’s *t* test (**p < 0.01, WT vs. Tg), or two-tailed Shirley-Williams test (^##^*p* < 0.01, Veh vs. 1 and 3 mg/kg in Tg mice). n = 8–10. Bar = 500 μm.

The potential therapeutic actions of T-518 were evaluated in the P301S tau Tg tauopathy mouse model. T-518 was administered at 1 and 3 mg/kg to 5-month-old P301S tau Tg mice for 2 weeks and axonal function was evaluated by observing the retrograde transport of Fluoro-Gold from hippocampus to medial septal nucleus as previously reported^36,37^. T-518 at 3 mg/kg significantly improved axonal transport in P301S tau Tg mice (Fig. 6).

### Suppression on insoluble tau by T-518 in a tauopathy mouse model

**Figure 7:**
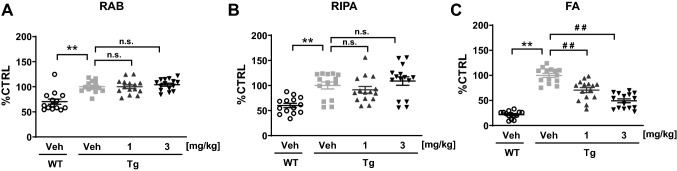
Mitigation of tau accumulation in P301S tau Tg mice by T-518. Six-month-old P301S tau Tg mice were treated with vehicle or T-518 (1 and 3 mg/kg) daily for 3 months. The hippocampus was isolated and RAB‒RIPA‒FA extraction was performed to assess the amount of insoluble tau by Western blotting and quantitatively analyzed. The original data of Western blotting is presented in Figure S4. Data are expressed as scatter plots and as mean ± SEM, and analyzed statistically as follows: Aspin-Welch *t* test (**p < 0.01, WT-Veh vs. Tg-Veh) or two-tailed Williams test (^##^*p* < 0.01, Tg-Veh vs. Tg-1 or 3 mg/kg). n = 14 or 15. *n.s.* not significant.

Moreover, T-518 was administered to P301S tau Tg mice from 6 to 9 months of age to evaluate effects on the observed age-dependent tau accumulation^38,39^ (Figures S3 and S7). Treatment with 3 mg/kg significantly decreased aging-dependent accumulation of tau in the FA fraction (i.e. RIPA-insoluble tau) in mouse hippocampus without affecting the RAB-soluble and RIPA-soluble fractions (Fig. 7 and Figure S4).

### Behavioral improvement by T-518 in a tauopathy mouse model

**Figure 8:**
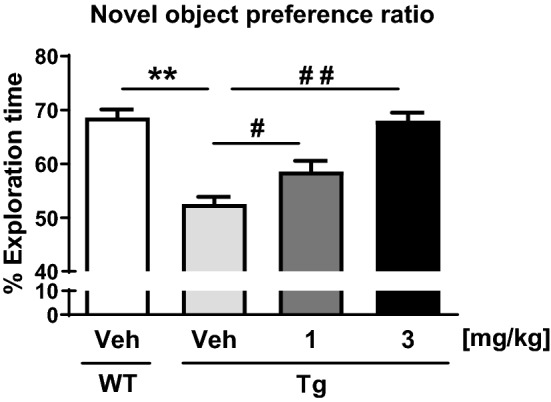
Restoration of impaired novel object recognition in P301S tau Tg mice by T-518. The novel object recognition test was conducted after 2 weeks of vehicle or T-518 administration (1 and 3 mg/kg). Novel object recognition performance is expressed as a preference ratio. Data are expressed as mean ± SEM and analyzed statistically as follows: Student’s *t* test (**p < 0.01, WT-Veh vs. Tg-Veh) or two-tailed Williams test (^#^*p* < 0.05 and ^##^*p* < 0.01, Veh vs. 1 and 3 mg/kg in Tg), n = 14 or 15.

Finally, we examined the potential benefits of T-518 treatment on hippocampus-dependent cognitive behavior using the novel object recognition test. Consistent with the demonstrated insoluble tau reduction, T-518 treatment dose-dependently ameliorated the novel object recognition deficit observed in vehicle-treated Tg mice compared to WT mice (Fig. 8).

## Discussion

In this study, we characterized the potency, target selectivity, PK, and bioactivity of the novel HDAC6 inhibitor T-518. In vitro, T-518 inhibits HDAC6 with an IC50 of 4.6 nM and demonstrates superior selectivity compared to the known HDAC6 inhibitors ACY-738, Ricolinostat, and Tubastatin A, as T-518 does not inhibit HDAC1‒5, 7‒9, and 11 up to 10 μM, while ACY-738 and Ricolinostat slightly inhibit class I and class IIa HDACs and Tubastatin A inhibits class IIa HDACs (Fig. 2A). This selectivity profile of HDA6 was also confirmed in mouse neurons by measuring the changes in HDAC substrate acetylation status (Fig. 2B–G). Treatment of T-518 increases tubulin acetylation but not Histone H3 acetylation at 0.01‒10 μM. Elevated tubulin acetylation was observed even at concentrations as low as 0.01 μM and statistical significance is detected at ≥ 0.1 μM. This high bioactivity is consistent with the low HDAC6 IC50 revealed by enzymatic assay. In contrast, Histone H3 acetylation is not enhanced by T-518, in contrast to ACY-738 and Ricolinostat treatment. Histone H3 is deacetylated by class I HDAC but not by HDAC6, so HDAC1‒3 inhibition could account for elevated H3 acetylation by ACY-738 and Ricolinostat. Therefore, T-518 is considered one of the best-in-class HDAC6 inhibitors.

Our in vivo PK study of T-518 demonstrated that T-518 has good CNS penetration with enough exposure to exert pharmacological activities. Administration of T-518 increases tubulin acetylation in the WT mouse brain but not in HDAC6 KO mice, indicating that HDAC6 is the principal molecular target. Further, the C_max_ and AUC_0–24 h_ in the hippocampus in the administration of 3 mg/kg are 144 ng/g and 202 ng·h/g, respectively (Table 1). Effective C_max_ and average exposure in 24 h are roughly correspond to 291 nM and 17 nM as free fraction in a brain (Fu,b) of T-518 is 0.4 (data not shown), and are greater than IC50. PK profile of a single oral dose is sharp: T_max_ is observed within 0.25 h and then, the concentration is rapidly decreased. On the other hand, the maximum pharmacodynamics change is observed at 1‒4 h after administration. This hysteresis may be explained by the slow off-rate of T-518 binding to HDAC6. Time dependent inhibition of T-518 in the enzyme assay (Fig. 1B) supports this hypothesis although there remains the possibility of slow turnover rate of acetyl tubulin to derive the hysteresis^40^. Sustained pharmacodynamics effect could be beneficial for chronic administration and for minimization of off-target effects in the clinical setting.

HDAC6 inhibitors are suggested to improve axonal transport of mitochondria or brain derived neurotrophic factor (BDNF) in diverse disease cell models^15,23–25^, and indeed T-518 also enhanced retrograde transport from hippocampus to medial septal nucleus in mice (Fig. 6). As axonal damage is a cardinal feature of tauopathy and deficient axonal transport has been reported in tau Tg mice^37,41–43^, we examined if T-518 could rescue this deficit in the P301S tau Tg mouse. Indeed, 2 weeks of oral treatment at 3 mg/kg significantly improved axonal transport in the septo-hippocampal pathway. This finding suggests that elevation of acetylated tubulin can improve axonal function in vivo. In some studies, axonal deficit was detected before NFT-like tau accumulation and brain atrophy, neither of which is robust in P301S tau Tg at the tested age. Tubulin Lys40 acetylation may thus be a major mechanism underlying this improvement in axonal transport by T-518. In fact, Lys40 acetylation was reported to promote the axonal association and motility of kinesin-1, a motor protein involved in axonal transport^44^. In turn, this improved cytoskeletal function may help preserve the structural stability of axons and enhance the delivery of trophic factors such as BDNF to the neuronal nucleus.

Oral administration of T-518 also reduces insoluble tau in mouse brain, consistent with previous studies suggesting that hydroxamate HDAC6 inhibitors can reduce pathological tau accumulation^26–29^. In addition, MT stabilizing agents have been reported to block disease progression in mouse models of tauopathy^45–47^, supporting our speculation that MT stabilization by HDAC6 inhibition could reduce tau accumulation and improve neural function, including hippocampus-dependent cognitive function, in tau Tg mice. As most MT stabilizing agents have been originally discovered as anti-cancer therapeutics, they may have cytotoxic effect based on its mechanism of action and have limitation of dosage. T-518 could be an alternative approach with wider therapeutic window compared to MT stabilizing agents although it was not unveiled why they have been unsuccessful in clinical trials. Chronic treatment with T-518 at 1 and 3 mg/kg significantly decreased RIPA-insoluble tau without affecting the amount of RAB-soluble or RAPA-soluble tau (Fig. 7). Selective effect of T-518 on RIPA-insoluble tau may be accounted for by change in subcellular localization of tau based on axonal stabilization.

While axonal abnormalities are an early pathological sign in both AD and tauopathy mouse models, and elevated tau induces axonal dysfunction in vitro and in vivo, axonal function can also influence tau accumulation^48^. Therefore, it is still unclear whether tau accumulation triggers axonal dysfunction or vice versa. It was reported that reduction of axonal transport by deletion of KLC1 resulted in tau accumulation^49,50^, suggesting that deficient axonal transport and tau accumulation mutually reinforcing. Our study indicates T-518 can rescue axonal function and thereby disrupt this vicious circle.

Here, our results indicate the therapeutic effect of T-518 in tauopathy mouse model, however the underlying mechanism of action has not been fully elucidated yet. There may be other mechanisms by which HDAC6 inhibition suppresses tau accumulation as HDAC6 is involved in many diverse biological pathways. For instance, HSP90 is relevant to proteasomal degradation of proteins and is also a HDAC6 substrate. Thus, HDAC6 inhibitors may promote tau degradation via the ubiquitin‒lysosomal pathway^51^. Also, acetylated tau can be a HDAC6 substrate and acetylation at the tau KXGS motif directly influences aggregate formation and degradation^52^. Future studies are warranted to investigate the effects of T-518 on HSP90 and tau acetylation. It is also known that HDAC6 has a ubiquitin-binding C-terminal domain that can recruit ubiquitinated protein aggregates to the aggresome^53–56^. Hence, aggresome formation or autophagosome maturation may contribute to tau reduction by HDAC6 inhibition.

Importantly, the recent report by Gamache and colleagues alerted that the potential genomic destruction by the P301L tau transgene contributes to tauopathy-like histopathological and behavioral abnormalities in the case of rTg4510, another tau Tg mouse line^57^. Although tau overexpression is closely linked to pathological changes in the line used in this study as proven by the amelioration under the treatment of ASO^58^, we have to pay attention to off-target effect in a study using Tg model. In addition, tau Tg mice are widely used for non-clinical pharmacological evaluation, however their translational value for clinical development could be limited. Additional studies with a different line may strengthen therapeutic potential of a drug candidate.

Finally, we also confirm that our HDAC6 KO mice are viable and normally developed similarly to the previously reported lines^59,60^, suggesting that selective inhibition of HDAC6 is unlikely to induce severe adverse events. In fact, chronic dosing was well tolerated by experimental mice.

In summary, T-518 is a novel HDAC6 inhibitor with a unique chemical structure, and superior potency and selectivity. Our observation supports that T-518 is a promising candidate for the treatment of AD and tauopathy. Further, T-518 has PK properties that may prove advantageous for other neurological disorders such as amyotrophic lateral sclerosis^61^, PD^62^, Charcot-Marie-Tooth disease^63^, and chemotherapy-induced peripheral neuropathy^64^.

## Methods

### HDAC enzyme assay and selectivity

Recombinant HDAC6, HDAC7, HDAC1 were obtained from SignalChem Pharmaceutical Inc. (Richmond, BC, Canada), and recombinant human HDAC4 was prepared at Takeda pharmaceutical company. One μL of T-518 diluted with DMSO was added to 75 μL of assay buffer (24 mM Tris–HCl (pH 7.5), 135 mM NaCl, 0.35 mM KCl, 1 mM MgCl_2_, 0.01% Tween 20, 0.6 mM GSH) in 384 well plates. Two μL of diluted compounds were added to 384 well plates and 2 μL of recombinant HDAC enzymes was added and then centrifuged at 1000 rpm for 10 s. The final concentrations of each HDAC enzyme were as follows, human HDAC1, 170 pM; human HDAC6, 3.0 M; human HDAC4, 68 pM; human HDAC7, 13 pM. After incubation for 60 min at room temperature, 4 μL of HDAC-Glo reagent [HDAC-Glo I/II (Promega) for HDAC6 and HDAC1, HDAC-Glo class IIa (Promega) for HDAC4 and HDAC7] was added to start the reaction. After incubation for 20 min at room temperature, the luminescence was measured by Envision plate reader (PerkinElmer, Waltham, MA, USA). For experiments under the condition of 0 min preincubation, 4 μL of HDAC-Glo reagent was added at first to the 384 well plates including diluted compounds. After centrifugation at 1000 rpm for 10 s, 2 μL of HDAC enzyme solution was added. After centrifugation at 1000 rpm for 10 s. and then incubation for 20 min at room temperature, the luminescence was measured by Envision. For determination of inhibitory activity of T-518, the vehicle signal was set as 0% and the signal in the absence of HDAC enzyme was set as 100% inhibition. The inhibitory activity of T-518 was expressed as the value of IC50. The IC50 value and 95% confidence intervals were calculated by XLfit (IDBS, Surrey, UK). The HDAC selectivity panel was conducted by Reaction Biology Corporation (Malvern, PA, USA).

### Animals

C57BL/6J mice were supplied by CLEA Japan Inc. (Tokyo, Japan) and HDAC6 KO mice were generated and supplied by Axcelead Drug Discovery Partners Inc. (Kanagawa, Japan). Human P301S mutant tau (4R1N) Tg mice were generated by the researchers in University of Pennsylvania^38^ and introduced to our facility. Male mice were used in all pharmacological examinations. All mice were housed in groups under a 12 h-light/12 h-dark cycle with ad libitum access to food and water. For sampling, animals were euthanized by exsanguination following decapitation. In anesthesia, medetomidine (Nippon Zenyaku, Fukushima, Japan), midazolam (Sandoz, Tokyo, Japan), and butorphanol (Meiji Seika Pharma, Japan) were intraperitoneally administered at 0.3 mg/kg, 4 mg/kg, and 5 mg/kg, respectively. Animals were maintained and sacrificed according to the guidelines of the Institutional Animal Care and Use Committee (IACUC) of Takeda Pharmaceutical Company Limited, which is accredited by the Association for Assessment and Accreditation of Laboratory Animal Care International (AAALAC). All animal experiments were performed in accordance with ARRIVE guidelines.

### Generation of HDAC6 knockout (KO) mice

HDAC6 knockout (KO) mice were generated by deletion of a ~ 1.7-kbp region including exon15–20 of the *Hdac6* gene using fertilized C57BL/6J eggs and the CRISPR-Cas9 system. We selected gRNA sequences for mouse *Hdac6* that do not have critical off-target candidates that cleave exons in other genes by CRISPRdirect (https://crispr.dbcls.jp/)^65^. Male hemizygous KO mice were selected by qPCR detection of the *Hdac6* intron16 sequence (Figure S2A). The qPCR primers and probe sequences were as follows: FW primer; 5′-GGCTGTGTGGATTTTAGGTTTTATG-3′, RV primer; 5′-CTGGATCCCATTACACTCTTGAGA-3′ and MGB probe; 5′-CCAGGCCATGTTTT-3′.

### Chemical treatment

T-518, ACY-738, Ricolinostat, and Tubastatin A were synthesized in Takeda. For in vitro cellular experiments, the compounds were solubilized with DMSO and diluted in the culture medium at the indicated concentrations. For in vivo experiments, the compound was reconstituted in 0.5% methylcellulose and administered orally at the indicated doses. After administration, mouse hippocampus was isolated and homogenized in RIPA extraction buffer (50 mM Tris–HCl, 150 mM NaCl, 1% NP-40, 0.5% sodium deoxycholate, and 0.1% SDS, pH 8.0) supplemented with protease and phosphatase inhibitor cocktail (Thermo Fischer, Waltham, MA, USA). The homogenate was centrifuged at 10,000*g* for 15 min and the supernatant was taken as the RIPA-soluble fraction.

### Antibodies

The following antibodies were used in this study: PathScan Total α-tubulin sandwich ELISA Antibody Pair (Cell Signaling Technology, Danvers, MA, USA), anti-Lys40 acetylated tubulin (Sigma, St. Louis, MO, USA), α-tubulin (B-5-1-2) (Sigma), anti-acetylated Histone H3 (EMD Millipore, Temecula, CA, USA), anti-Histone H3 (Cell Signaling Technology), anti anti-HDAC6 (D21B10) (Cell Signaling Technology), and anti-actin (AC-15) (Sigma), anti tau (Thermo Fisher).

### Mouse primary neural culture

Primary cortical neurons were prepared from E14 C57BL/6J mouse embryos (CLIEA Japan, Inc., Tokyo, Japan) using papain-containing Neuron Dissociation Solutions (Fujifilm Wako, Tokyo, Japan). Isolated cells were suspended in Neurobasal Medium containing B-27 (Thermo Fisher) and seeded on poly-d-lysine-coated plates (Corning, NY, USA) under 5% CO_2_ at 37 °C for 6 days in vitro (DIV) prior to evaluation of HDAC6 inhibitors. Cells were treated with HDAC6 inhibitors at the indicated concentrations for 24 h and lysed in RIPA extraction buffer (50 mM Tris–HCl, 150 mM NaCl, 1% NP-40, 0.5% sodium deoxycholate, and 0.1% SDS, pH 8.0) supplemented with protease and phosphatase inhibitor cocktail (Thermo Fisher).

### Measurement of acetylated α-tubulin and total tubulin

Acetylated α-tubulin and total tubulin levels were measured by sandwich ELISA. Ninety six-well plates (Microlite2+, Thermo) were coated with α-tubulin capture antibody (1:100) by overnight incubation at 4 °C, washed 4 times with PBS'T, treated with blocking buffer (1% bovine serum albumin in PBS'T) at 37 °C for 2 h, and washed another 4 times with PBS’T. Cell or tissues lysate was added to the wells and plates were incubated at 37 °C for 2 h. Plates were then washed 4 times with PBST, incubated with a detection antibody for acetylated tubulin or total α-tubulin diluted in blocking buffer at 37℃ for 1 h, rewashed 4 more times with PBST, incubated with a mouse secondary antibody conjugated to horseradish peroxidase (HRP) for 30 min, wash 4 times again with PBST, and then incubated with chemiluminescent substrate (SuperSignal).

### Measurement of HDAC6 inhibitors by LC–MS/MS

The compound concentration was measured by the method previously reported with modification^66^. Briefly, the hippocampus tissues from treated mice were isolated and homogenized in 4 volumes of saline under ice-cold conditions and stored at − 80 °C until liquid chromatography-tandem mass spectrometry (LC–MS/MS) analysis. Before analysis, samples were thawed gently on ice and mixed with three volumes of acetonitrile. The acetonitrile mixtures were vortexed and centrifuged for supernatant collection. The supernatant was diluted in mobile phase A [10 mM ammonium formate and formic acid at 100:0.2 (v/v)] and injected into the LC/MS/MS system. T-518 was analyzed in multiple reaction monitoring mode using an API4000 or API5000 instrument (Applied Biosystems, Foster City, CA, USA) equipped with a Prominence Ultra-fast liquid chromatography system (Shimadzu, Kyoto, Japan) using Shimadzu Shim- pack XR-ODS (2.2 μm, 2.0 × 30 mm) column. Mobile phase B consisted of a mixture of acetonitrile and formic acid at 100:0.2 (v/v). Chromatographic separations were performed by gradient elution at a flow rate of 0.7 mL/min. Mobile phase B was held at 5% for 0.2 min, increased linearly to 99% for 1.1 min, maintained at 99% for 0.7 min, then returned to 5% in 0.01 min, followed by re-equilibration for 0.59 min. A multiple reaction monitoring was used to detect the parent and product ions for the tandem mass spectrometry of T-518, which were corresponding to *m/z* 494 and *m/z* 374, respectively, with the negative ion mode [DP; -40, EP; -10, CE; -35, CXP; -15 (arbitrary units)]. Analyst software TM (version 1.6.2) (AB SCIEX, Framingham, MA; https://sciex.com/products/software/analyst-software) was used for data acquisition and processing.

### Western blotting

Cell lysate or in vivo protein samples were separated by 10% SDS-PAGE, then electrophoretically transferred to 0.45 μm poly-vinylidene difluoride membranes (Millipore). Membranes were blocked for 1 h in Blocking Reagent (TOYOBO, Osaka, Japan), probed with primary antibodies followed by labeling with HRP-coupled secondary antibodies (Amersham, Piscataway, NJ), and then visualized using chemiluminescence reagent (ECL; Amersham). Images were captured using ImageQuant LAS 4000 (GE healthcare Bio-Sciences, Marlborough, MA, USA). Quantitative densitometric analyses were conducted using ImageQuant TL (GE healthcare Bio-Sciences). The protocol was slightly modified for detection of HDAC6 in cortex. Briefly, cerebral cortex was homogenized in RIPA buffer supplemented with 1 μM microstatin, 1 μM MG115, 40 μM Leupeptin, 100 μM ABSF, and 2 mM sodium orthovanadate, separated by 7.5–15% gradient SDS-PAGE and electrophoretically transferred to 0.45 μm poly-vinylidene difluoride membranes (Millipore). Membranes were blocked for 1 h with ECL Prime Blocking Reagent (Amersham, Piscataway, NJ) prior to immunoblotting, visualization, and densitometry as described above.

### Evaluation of axonal transport

The integrity of retrograde axonal transport was evaluated by detecting the accumulation of Fluoro-Gold (Fluorochrome, Denver, CO) in the medial septal nucleus after injection into hippocampus (0.5 μL of 5% at AP − 2.3 mm, ML − 2.0 mm, and DV − 2.0 mm relative to bregma). Three days after injection, mice were anesthetized and transcardially perfused with PBS (pH 7.4) followed by 4% paraformaldehyde. Fixed brains were embedded and frozen in freezing medium and sectioned at 20-μm thickness using a cryostat as described above. Sections were mounted onto silane-coated slides and coverslipped with mounting medium (VECTASHIELD; VECTOR, Burlingame, CA). Images were captured using a microscope (ECLIPS E800M; Nikon, Tokyo, Japan) and a camera (DXM1200, Nikon). Fluorescent cells in the medial septum region were counted with Image-Pro Plus software (version 4.5.0.24) (Media Cybernetics, Rockville, MD; https://www.mediacy.com/imageproplus).

### Isolation of tau fractions by sequential RAB‒RIPA‒formic acid (FA) extraction

Tau fractions were separated by sequential RAB-RIPA-FA extraction as described^67,68^. Brain tissue was homogenized in ice-cold high-salt RAB buffer [0.1 M morpholineethanesulfonic acid (MES), 1 mM EGTA, 0.5 mM MgSO_4_, 0.75 M NaCl, 0.02 M NaF, 100 μM ABSF; pH 7.0] supplemented with protease and phosphatase inhibitor cocktail (Thermo Fisher). Sample was centrifuged at 50,000×*g* for 20 min at 4 °C, and the supernatant was boiled for 5 min, and recentrifuged at 10,000×*g* for 20 min at 4 °C. This second supernatant contains the soluble tau fraction (RAB fraction). To remove myelin and associated lipids, RAB insoluble pellets were resuspended with 1 M sucrose/RAB buffer and centrifuged at 100,000×*g* for 30 min at 4 °C. The pellets were suspended in RIPA buffer (50 mM Tris–HCl, 150 mM NaCl, 1% NP-40, 0.5% sodium deoxycholate, and 0.1% SDS, pH 8.0) supplemented with protease and phosphatase inhibitor cocktail (Thermo Fisher) and recentrifuged as above to obtain the supernatant (RIPA fraction). Finally, the RIPA insoluble pellets were extracted with 70% FA (FA fraction).

### Behavioral assessments

The effect of T-518 on behavioral function was examined using the novel object recognition test: The box and objects used were purchased from BrainScience Idea (Osaka, Japan). The 30 × 30 × 35 cm gray-colored test box was used. The two objects used were pyramidal, white-colored, and ceramic (object A and A′) and cylindrical, silver, and aluminum (object B). In the acquisition phase, the identical objects (objects A and A′) were symmetrically placed in the box. Each animal was placed in the box for 5 min followed by a 24-h retention interval in the home cage. Mice were placed at the corner of the box turning their heads toward the wall. The test box and objects were cleaned with 10% ethanol between trials. After replacing one of the objects with a novel object (object B), the mouse was reintroduced into the box for a 5-min retention phase. Time spent exploring the objects were measured during both phases. Retention was represented by how long the animals explored the novel object in the total exploration (Novel object preference ratio).

### Statistical analysis

Statistical analysis was performed using EXSUS (CAC EXICARE Corporation, Tokyo, Japan). For comparison of two groups such as WT vs. Tg mice, Bartlett's test was performed to assess the homogeneity of variances, followed by Student’s *t* test (for parametric data: *p* > 0.05 by Bartlett’s test) or Aspin-Welch *t* test (for non-parametric data: *p* < 0.05 by Bartlett’s test). For multiple comparison, Bartlett's test was performed to assess the homogeneity of variances, followed by Tukey test (for parametric data: *p* > 0.05 by Bartlett’s test) or Steel–Dwass test (for non-parametric data: *p* < 0.05 by Bartlett’s test). In the dose- or concentration–response studies, Bartlett's test was performed to assess the homogeneity of variances, followed by two-tailed Williams’ test (for parametric data: *p* > 0.05 by Bartlett’s test) or two-tailed Shirley-Williams test (for non-parametric data: *p* < 0.05 by Bartlett’s test) to compare multiple doses of the test compounds to the control group in a dose-dependent study. Differences yielding *p* values < 0.05 were considered significant.

### Approval for animal experiments

All research involving the animals has been approved by the IACUC (Institutional Animal Care and Use Committee) of Takeda Pharmaceutical Company Limited, accredited by AAALAC (Association for Assessment and Accreditation of Laboratory Animal Care International).

## Supplementary Information

Supplementary Figures.

## Data Availability

All raw data and materials are available on request.
