# Supplementary material for: A novel orally active HDAC6 inhibitor T-518 shows a therapeutic potential for Alzheimer’s disease and tauopathy in mice
Source: Sci Rep. 2021 Jul 29;11:15423. doi: 10.1038/s41598-021-94923-w (PMC8322070; doi:10.1038/s41598-021-94923-w)
Supplement: Supplementary file 1 — Supplementary Figures. [file 41598_2021_94923_MOESM1_ESM.pdf]

Supplementary Information for

A Novel Orally Active HDAC6 Inhibitor T-518 Shows a Therapeutic Potential for Alzheimer's Disease and Tauopathy in Mice

Tomohiro Onishi*, Ryouta Maeda, Michiko Terada, Sho Sato, Takahiro Fujii, Masahiro Ito, Kentaro Hashikami, Tomohiro Kawamoto, and Maiko Tanaka

* Correspondence: tomohiro.onishi@takeda.com

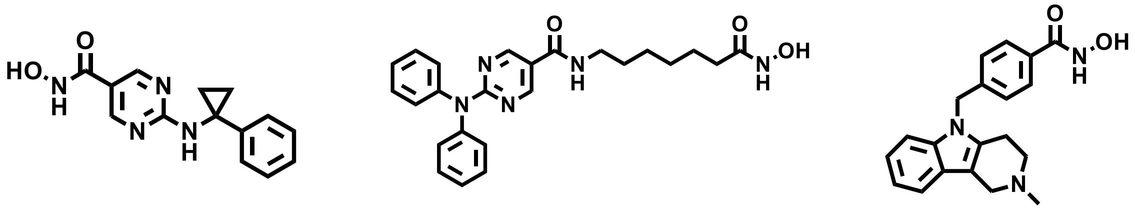

Figure S1

Chemical structures of hydroxamate HDAC6 inhibitors ACY-738, Ricolinostat, and Tubastatin A

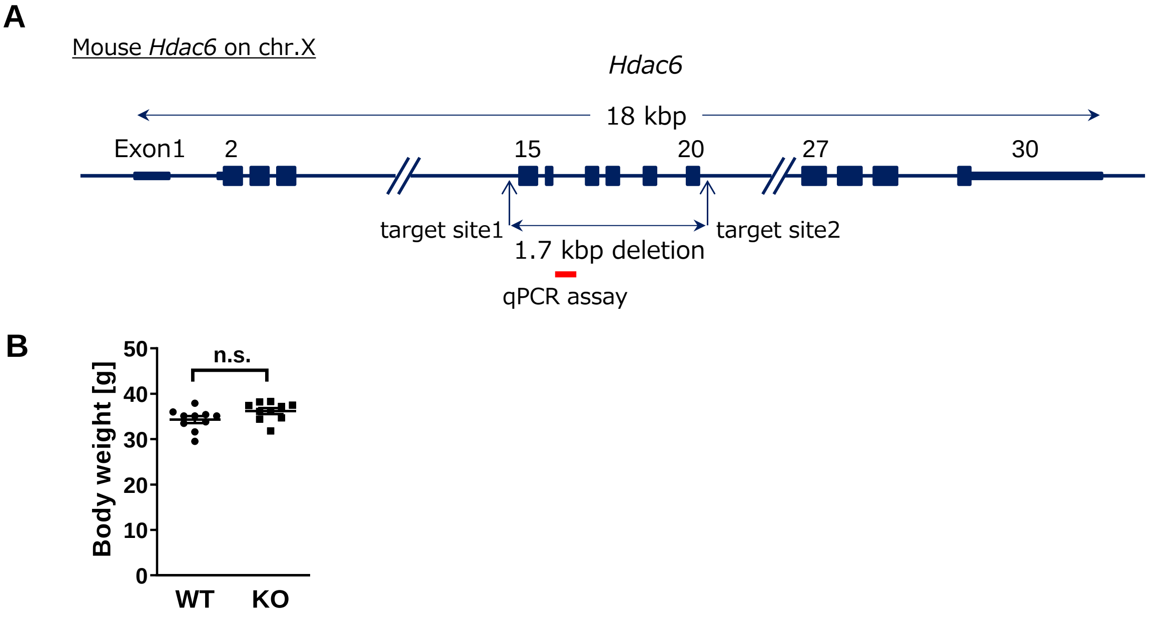

Figure S2

Generation of Hdac6 knockout mouse and comparison of body weight between WT and KO

Strategy for generation of Hdac6 knockout mouse is illustrated (A). Target sequences for Hdac6 exon15 - 20 deletion were as follows: 5'-GTAAGTAGGCAACGTGCTAA-3' for target site 1 and 5'-CCCAAAACCCGATCTTTGCA-3' for target site 2. Body weights of male WT and KO mice at the age of 27 weeks were measured (B).

Data are expressed as mean ± SEM and as scatter plots, and analyzed statistically a follow: Student’s *t*-test. n = 10. n.s., not significant.

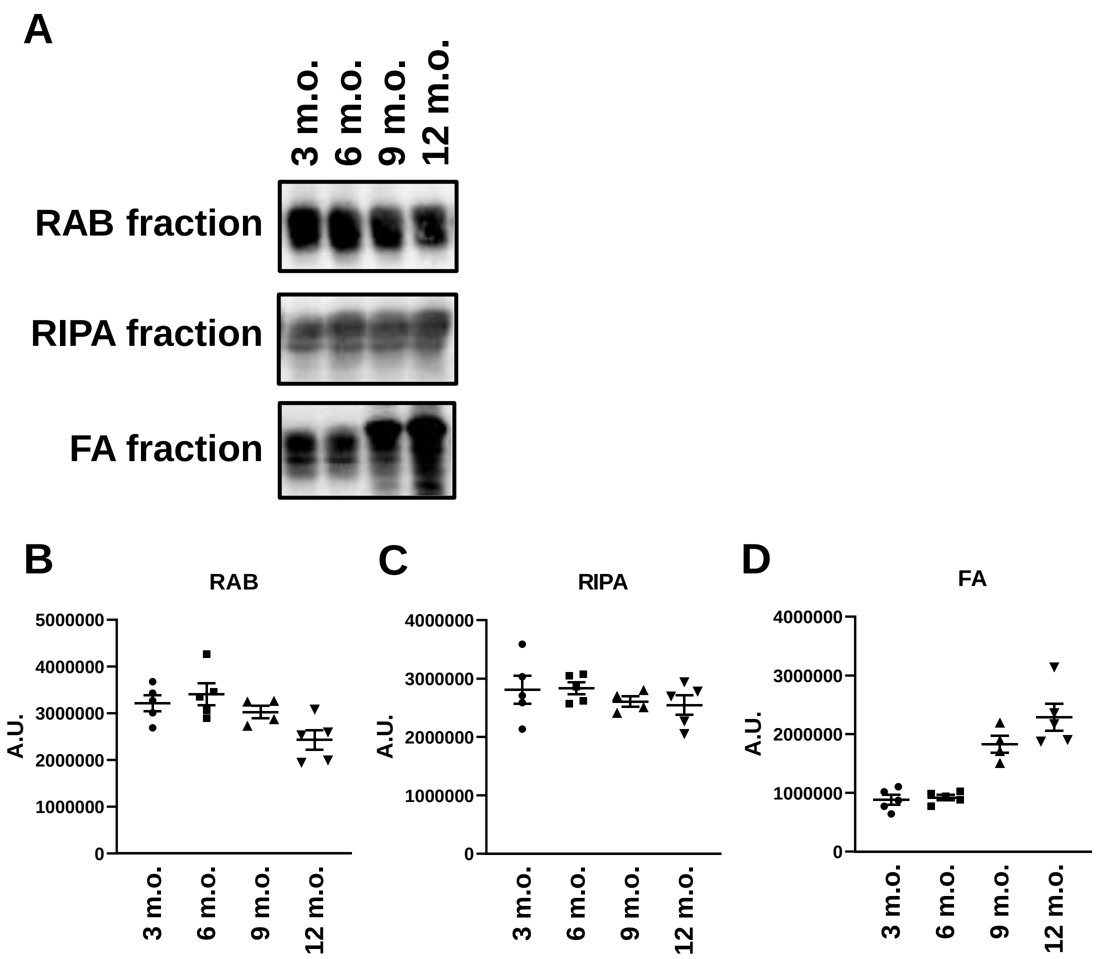

Figure S3

Age dependent change of tau in P301S tau Tg mouse

Hippocampi were obtained from male, 3-, 6-, 9-, and 12-month-old P301S tau Tg mice and RAB-soluble, RIPA-soluble, and FA-soluble tau was evaluated by Western blotting (A) and quantitatively analyzed (B-D). Full-length blot is presented in Figure S7.

Data was expressed as mean± SEM and as scatter plots (B-D). Statistical analysis was not performed.   n = 4 or 5.

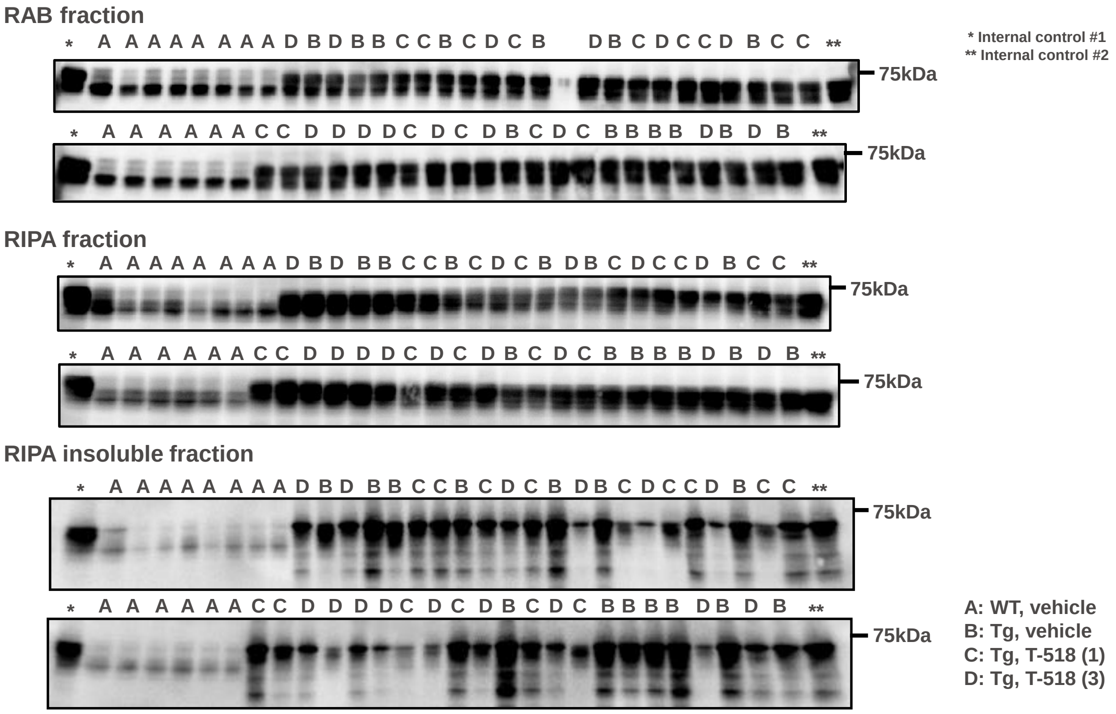

Figure S4

Individual data of Figure 7

SDS-PAGE of each fraction was examined in the separate gels because of the number of lanes. Immunoblotting was proceeded in parallel and quantitative comparisons between images were conducted under normalization with the internal controls.

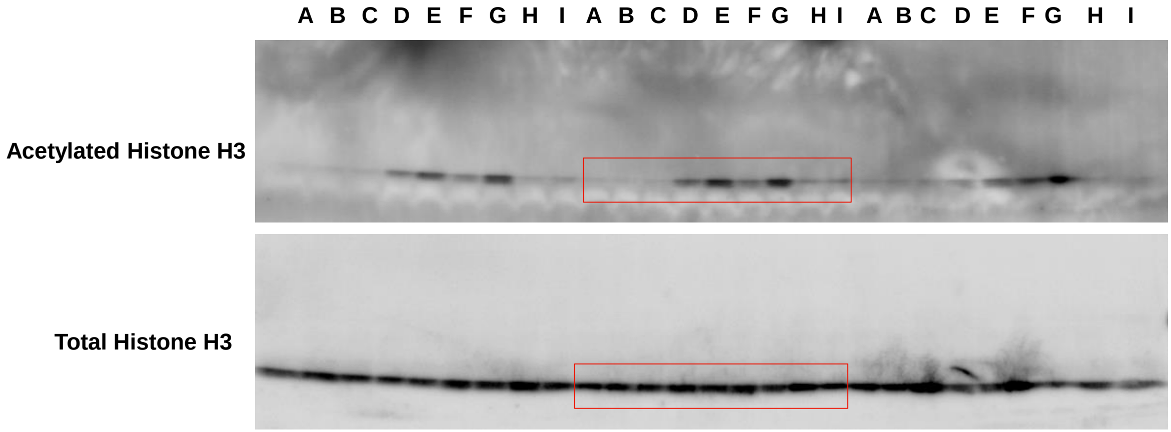

A, DMSO
B, T-518 (3)
C, T-518 (30)
D, ACY-738 (3)
E, ACY-738 (30)
F, Ricolinostat (3)
G, Ricolinostat (30)
H, Tubastatin A (3)
I, Tubastatin A (30)

Figure S5

Individual data of Figure 2F

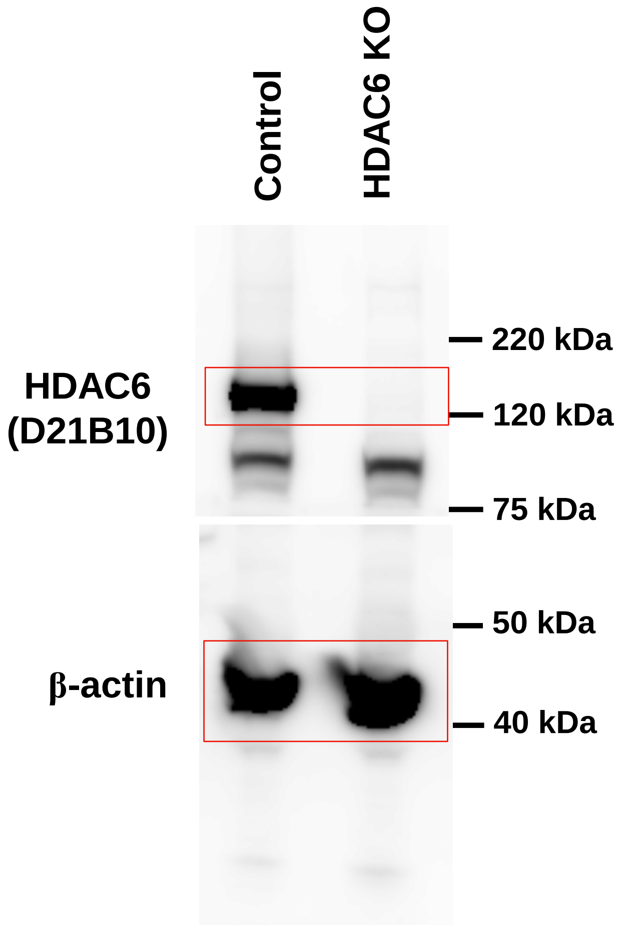

Figure S6
Raw data of Figure 5

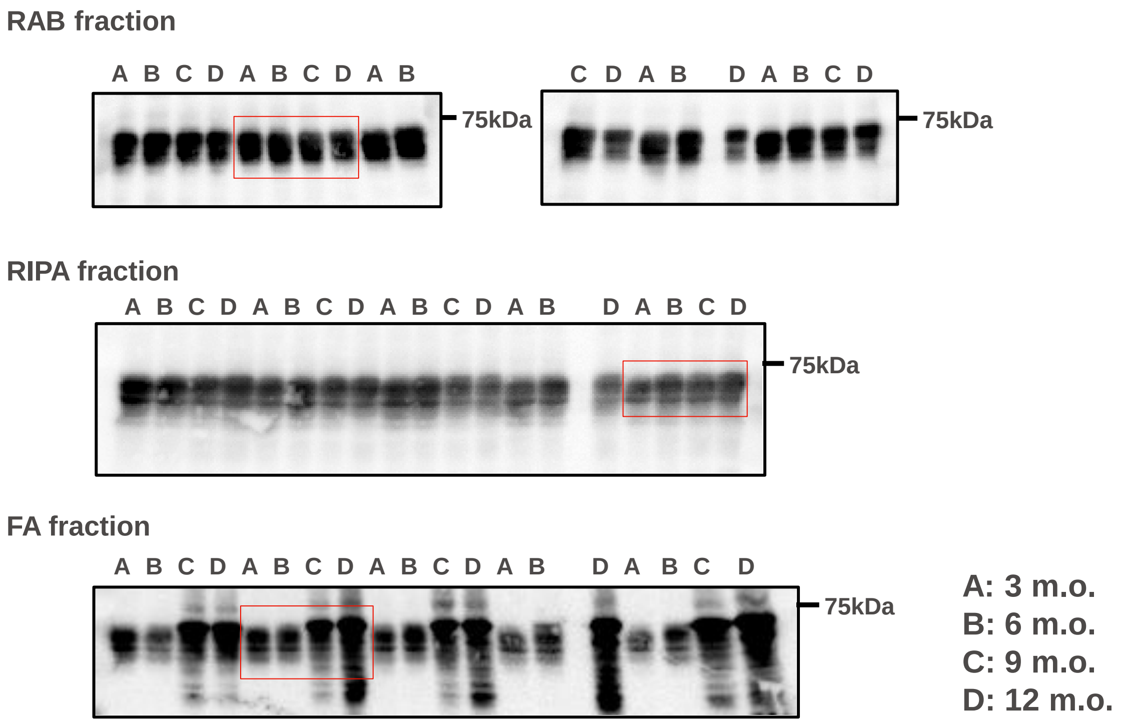

Figure S7

Raw data of Figure S3A

SDS-PAGE of RAB fraction was examined in the separate gels because of the number of lanes. Immunoblotting and imaging were processed in parallel.
